# BF_2_-Functionalized Benzothiazole Amyloid Markers: Effect of Donor Substituents on One- and Two-Photon Properties

**DOI:** 10.1021/acsabm.3c00815

**Published:** 2023-12-07

**Authors:** Agata Hajda, Manuela Grelich-Mucha, Patryk Rybczyński, Borys Ośmiałowski, Robert Zaleśny, Joanna Olesiak-Bańska

**Affiliations:** † Faculty of Chemistry, Wroclaw University of Science and Technology, Wybrzeże Wyspiańskiego 27, PL-50-370 Wroclaw, Poland; ‡ Faculty of Chemistry, Nicolaus Copernicus University, Gagarina Street 7, Toruń PL-87-100, Poland

**Keywords:** fluorescence probes, amyloids, two-photon absorption, BODIPY, thioflavin T

## Abstract

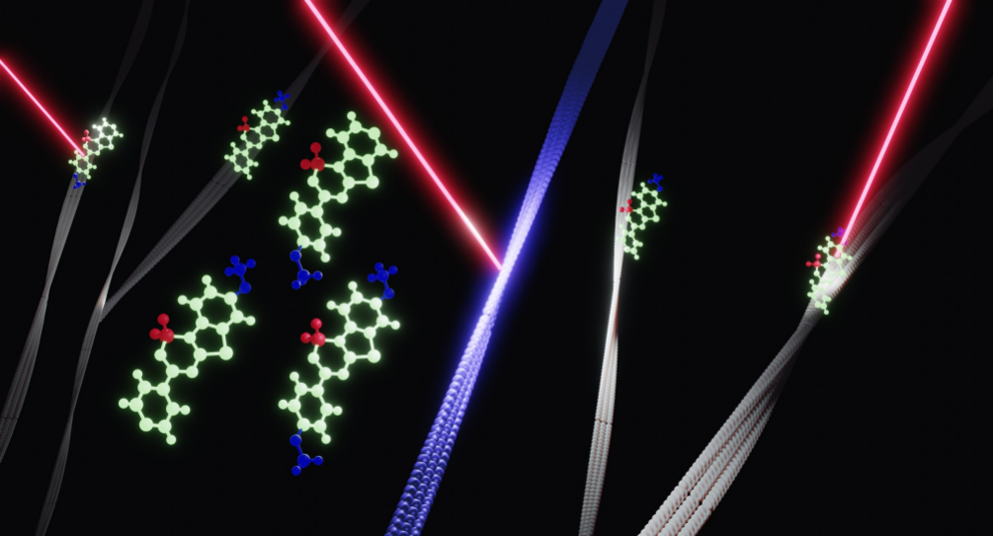

Investigation of amyloids with the aid of fluorescence microscopy provides crucial insights into the development of numerous diseases associated with the formation of aggregates. Here, we present a series of BF_2_-functionalized benzothiazoles with electron-donating methoxy group(s), which are tested as amyloid fluorescent markers. We evaluate how the position of donor functional group(s) influences optical properties (fluorescence lifetime (τ) and fluorescence quantum yield (FQY)) in a solution and upon binding to amyloids. We elucidate the importance of surrounding environmental factors (hydrogen-bonding network, polarity, and viscosity) on the observed changes in FQY and evaluate how the localization of a donor influences radiative and nonradiative decay pathways. We conclude that a donor attached to the benzothiazole ring contributes to the increment of radiative decay pathways upon binding to amyloids (*k*_r_), while the donor attached to the flexible part of a molecule (with rotational freedom) contributes to a decrease in nonradiative decay pathways (*k*_nr_). We find that the donor–acceptor–donor architecture allows us to obtain 58 times higher FQY of the dye upon binding to bovine insulin amyloids. Finally, we measure two-photon absorption (2PA) cross sections (σ_2_) of the dyes and their change upon binding by the two-photon excited fluorescence (2PEF) technique. Measurements reveal that dyes that exhibit the increase/decrease of σ_2_ values when transferred from highly polar solvents to CHCl_3_ present a similar behavior upon amyloid binding. Our 2PA experimental values are supported by quantum mechanics/molecular mechanics (QM/MM) simulations. Despite this trend, the values of σ_2_ are not the same, which points out the importance of two-photon absorption measurements of amyloid–dye complexes in order to understand the performance of 2P probes upon binding.

## 1 Introduction

Amyloids are misfolded proteins with a characteristic fibrillar morphology and a β-sheet-rich secondary structure. According to one of the hypotheses, their presence in tissues is one of the hallmarks of over 50 diseases, including various neurodegenerative disorders, such as Parkinson’s disease (PD) and Alzheimer’s disease (AD), and type 2 diabetes (T2D).^1^ The mentioned pathologies are incurable, and their etiology is still not fully understood.^2^ Accurately tracking the development and localization of amyloids inside cells is crucial for understanding disease progression and the development of therapeutic agents. Techniques such as magnetic resonance imaging (MRI),^3^ positron emission tomography (PET),^4^ and fluorescence microscopy enable the detection of amyloids *in vivo*.^5^ Fluorescence microscopy attracts broad attention, mainly due to its simplicity and higher spatial resolution compared to other mentioned techniques. Another type of microscopy, widely used *in vivo*, is two-photon microscopy (2PM). 2PM enables excitation in the near-IR region (>700 nm), which is beneficial due to its deeper tissue penetration, low phototoxicity, minimalized photobleaching, and imaging of small objects.^6,7^ 2PM has already been successfully employed in the visualization of Aβ amyloids located in the deep brain region.^8,9^ However, fluorescence microscopy requires fluorescent amyloid-binding agents to obtain a high signal-to-noise ratio. From the spectroscopic point of view, perfect markers should present the following features: (1) increase in FQY upon amyloid binding and/or shift in the maximum emission/absorption wavelength upon binding to differentiate between a dye in the bound and unbound states; (2) high photostability; and (3) emission and absorption in the first biological window (700–950 nm). When it comes to markers for 2PM, a high value of the product of two-photon absorption cross section (σ_2_) and FQY (ϕ), σ_eff_ = σ_2_ × ϕ, is additionally required. The latter quantity (σ_eff_) is also termed the two-photon action cross section.

**Figure 1:**
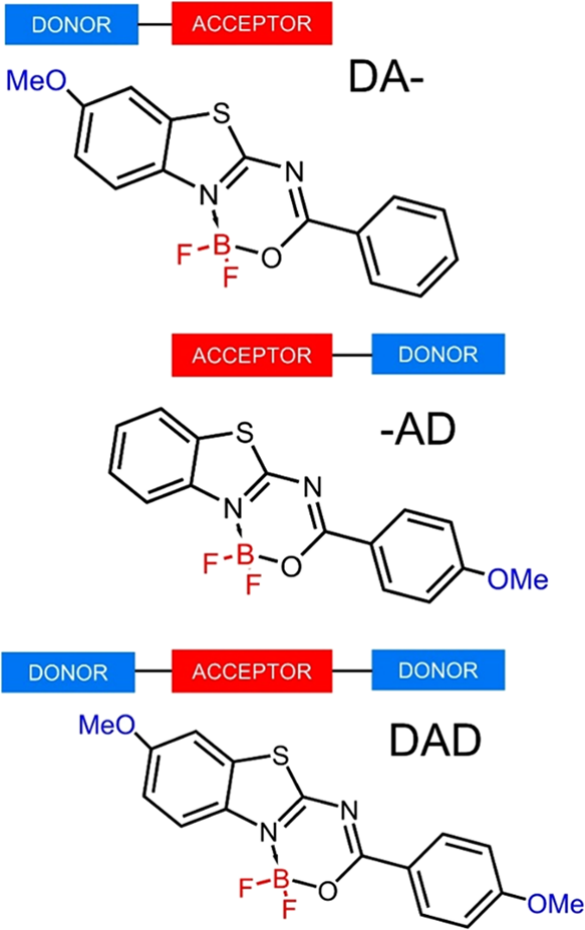
Structures of the investigated dyes.

The smart construction of new fluorescent dyes for amyloid detection is a challenging process. Most existing probes are based on popular fluorophores: benzothiazoles,^10−12^ BODIPY,^13−15^ curcumin,^16,17^ and thiophene.^18−20^ Moreover, various mechanisms of enhancing FQY of probes upon binding to amyloids are explored: (1) restriction of rotation of structural parts of a dye upon binding, which occurs in molecular rotors such as thioflavin T (ThT),^21^ (2) transition from a hydrophilic to a hydrophobic environment (e.g., Acedan derivatives),^22^ (3) dual mechanism of restriction and environment (e.g., ANCA),^23^ or (4) aggregation-induced emission.^24^ Recently, our group has synthesized a series of BF_2_-functionalized dyes with structural core inspired by ThT.^25^ By and large, dyes carrying the BF_2_ moiety attracted the attention of the scientific community as fluorescent probes due to their outstanding photophysical properties: high FQY, large molar extinction coefficients, tunable emission wavelength and narrow emission bands, high chemical stability, and high two-photon absorption cross sections.^26−28^ The difluoroborate (BF_2_) moiety acting as an electron acceptor can lead to strong intramolecular charge transfer (ICT) when combined with the electron donor group. The same moiety is also responsible for the rigidification of the molecular skeleton. The largest group of BF_2_-containing dyes is 4,4-difluoro-4-bora-3a,4a-diaza-s-indacenes (BODIPYs). In BODIPYs, two nitrogen atoms coordinate with an atom of boron; however, in some similar derivatives, nitrogen is replaced by oxygen. It is worth mentioning that N,O-coordinated BF_2_ probes were already used to detect Aβ plaques and neurofibrillary tangles in 1PM and 2PM, but there were no systematic studies of two-photon properties of probes before and after amyloid binding.^29,30^ Examination of multiphoton properties can provide new information about the relationship between the structure of the probe and amyloid interactions, which might differ from one-photon optical changes. In this paper, we investigate the potential of BF_2_-functionalized benzothiazoles with electron-donating methoxy group(s) as amyloid fluorescent markers. To gain systematic knowledge about the relationship between the dye structure, its optical properties, and their modification by binding to amyloids, we investigate three dyes with a weak electron donor located on one or both terminals of the fluorescent core (see Figure 1): (1) **DA-** ((*Z*)-[(difluoroboryloxy)phenylmethylene](6-methoxy-1,3-benzothiazol-2-yl)amine), (2) -**AD** ((*Z*)-[(difluoroboryloxy)(4-methoxyphenyl)methylene]-1,3-benzothiazol-2-ylamine), and (3) **DAD** ((*Z*)-[(difluoroboryloxy)(4-methoxyphenyl)methylene](6-methoxy-1,3-benzothiazol-2-yl)amine). Using the same benzothiazole core, it was possible to modulate the FQY between 0.4 and 98%^25^ by changing the position of the donor and acceptor units only. In the current study, changes in optical properties such as the fluorescence lifetime (τ), FQY, 2PA, 1P, and 2P excited fluorescence upon binding to amyloid fibrils are investigated. The discussion is focused on differences in the architecture of dyes, as well as different dependencies of FQY on surrounding environmental factors (hydrogen-bonding network, polarity, and viscosity) and the performance of dyes upon binding to amyloids. We indicate general principles in the evaluation of one- and two-photon optical properties of fluorescent amyloid markers. That is mainly based on their σ_2_ or σ_eff_ in moderate polarity media such as EtOH, DMF, and DCB, as the core of amyloids is similar in polarity to the mentioned solvents. Although scarcely presented in the literature, investigations of dyes bound to biopolymers such as DNA or proteins show that the orientation and localization of fluorophores inside biomolecules influence their 2PA cross sections.^31,32^ Thus, 2PA cross-sectional determination of free dyes in organic solvents may not be sufficient to predict their two-photon absorption upon binding to amyloids, which are complex biopolymers. Here, we evaluate the need for the determination of σ_2_ not only in solution but also after binding to amyloids for a reliable discussion on the structure–property relation in the design of two-photon amyloid markers.

## 2 Experimental Section

### 2.1 Synthesis of Dyes

The synthesis of dyes was described in our previous article.^1^

#### 2.2 Incubation of Bovine Insulin Amyloids

Bovine insulin (BI) was purchased from Sigma-Aldrich (I5500) and dissolved in HCl solution (pH ∼ 1.5), yielding the final concentration of 2 mg/mL. The samples were incubated in an Eppendorf ThermoMixer C for 40 h at 45 °C, with agitation set to 700 rpm.

#### 2.3 Incubation of the Hen Egg White Lysozyme (HEWL) Amyloids

HEWL was purchased from Sigma-Aldrich (L6876) and dissolved in HCl solution (pH ∼ 1.5) to yield a final concentration of 20 mg/mL (1.4 mM). The samples were incubated in an Eppendorf ThermoMixer C for 20 h at 85 °C, with the agitation set to 1400 rpm.

#### 2.4 Atomic Force Microscopy (AFM)

The full procedure is published elsewhere.^44^ In brief, samples were diluted to 0.01 mg/mL. The droplets of the samples were deposited on a mica layer, rinsed with Milli-Q water after 5 min, and dried afterward. Measurements were conducted by using a Veeco Dimension V atomic force microscope in tapping mode with the SuperSharpSilicon probe mounted (Manufacturer: NANOSENSORS).

#### 2.5 Absorption and Fluorescence Spectroscopies

One-photon absorption spectra were measured with a Jasco V-670 spectrophotometer in quartz cuvettes within the range of 280–700 nm. Stock solutions of dyes were prepared by dissolution in DMSO (500 μM), and all solutions were prepared before use. For samples of mixtures of water and DMSO, the appropriate volume of the stock solution was withdrawn and diluted so the volume of DMSO reached 40%; then, Milli-Q water was added. For samples with amyloids, the order of addition to prepare samples was as follows: DMSO, the stock solution of dye, the stock solution of amyloids, and Milli-Q water. The final concentration of the dye in fluorescence enhancement measurements upon amyloid binding was 2.5 μM. Fluorescence emission and excitation spectra were recorded using an FS5 Spectrofluorometer (Edinburgh Instruments) equipped with a xenon lamp.

#### 2.6 Selectivity toward Biomolecules and Bovine Serum Albumin (BSA)

Measurements of fluorescence changes of dyes (2.5 μM) upon binding to various bioanalytes and BSA were measured on a clarioSTAR Plus plate reader in a 96-well black plate. Fluorescence spectra were measured 10 min after the incubation of dyes with biomolecules at room temperature. To compare changes before and after binding, the fluorescence intensity (FI) at maximum wavelength before and after binding in the same solvent were divided by each other (FI_after binding_/FI_dye alone_).

#### 2.7 Fluorescence Quantum Yield

The FQY was measured by using the SC-30 Integrating Sphere Module for an FS5 spectrofluorometer from Edinburgh Instruments. The concertation of dyes was set to obtain a high signal on a two-photon microscope, as the FQY is used to calculate the 2PA cross section. For measurements before and after amyloid binding, the DMSO content was the same as that described in Section 2.5. The final concentration of dyes with and without amyloids was 2.5 μM. The amyloid concentrations were 122 μM for **DA-**, 82 μM for **-AD**, and 82 μM for **DAD**. These concentrations provided the best enhancement in fluorescence.

#### 2.8 Fluorescence Lifetime Characterization

One-photon excited fluorescence decays were acquired by time-correlated single-photon counting (TCSPC), the setup containing an Acton SpectraPro SP-2300 monochromator (Princeton Instruments) and a high-speed hybrid detector HPM-100-50 (Becker & Hickl GmbH) controlled by a DCC-100 card. As an excitation source, a BDL-375-SMN picosecond laser diode (20 MHz, λ_exc_ = 377 nm) was used. For each measurement, 6 scans were performed, which were later fitted in SPCImage software. For the fitting of data, synthetic IRF was used. The mean value of the fluorescence lifetimes from 6 scans was calculated to obtain the most reliable result. The mean value was used as the “real” data in the calculation of *k*_nr_ and *k*_r_. Decays were measured at λ_em_ = 430 nm (**-AD**), λ_em_ = 490 nm (**DA-**), and λ_em_ = 475 nm (**DAD**). Samples had the same concentrations of dyes and amyloids as those in FQY measurements.

#### 2.9 Calculation of *k*_r_ and *k*_nr_

To calculate *k*_r_ and *k*_nr_, the fitted average lifetime and measured FQY were used, as described in eqs 1 and 2
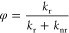
1
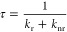
2

#### 2.10 Characterization of Nonlinear Optical Properties

Two-photon excited luminescence was measured using a custom-built multiphoton microscope consisting of a femtosecond mode-locked Ti:sapphire laser (∼100 fs, 80 MHz, Chameleon, Coherent Inc.) with an incident wavelength range tunable within λ = 700–1050 nm. Luminescence was recorded through a microscope objective (Nikon Plan Fluor, 40×, NA 0.75), and 2PEF signals were recorded in the epifluorescence mode. 2PEF spectra were measured with a Shamrock 303i spectrometer (Andor) equipped with an iDus camera (Andor). Samples and references were illuminated with the output power set between 40 and 70 mW depending on the measured dye (reference always was measured with the same power as the sample). Experimental conditions were chosen to prevent photobleaching and achieve a high signal-to-noise ratio. Two-photon absorption cross sections were calculated with eq 3
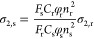
3

Effective two-photon absorption cross sections were calculated with eq 4
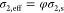
4where *C* is the fluorophore molar concentration per cubic centimeter, *n* the refractive index of the solvent, φ the fluorescence quantum yield, and *F* the integral over the whole two-photon excited emission band. The letters s and r correspond to the sample and reference, respectively. The chosen reference was a fluorescein solution in 0.1 M NaOH. The two-photon absorption cross section of fluorescein was obtained from elsewhere.^45^ Samples prepared in CHCl_3_ and H_2_O/DMSO before and after amyloid binding had the same concentrations of dyes and amyloids as in FQY measurements.

#### 2.11 Power Dependence of the Fluorescence Intensity

To confirm that the observed fluorescence excited by laser pulses had a two-photon nature, we measured the intensity versus excitation power dependence and determined the power exponent *n* (eq 5).
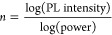
5where the PL intensity is a 2P excited photoluminescence intensity, and the power is the average incident laser power.

#### 2.12 Computer Simulations

The studied dyes were solvated with chloroform or water molecules, resulting in spherically symmetric clusters. Two-layer ONIOM calculations were performed for the clusters.^46^ The composition of layers was determined based on the following criteria:
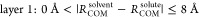

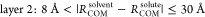
where *R*_COM_^*i*^ refers to the position vector of the center of mass (COM) of the *i*-th molecule. Subsequently, the optimization of the geometry was performed using GAUSSIAN program.^47^ In so doing, layer 1 was described at the B3LYP/6-31+G(d) level of theory with D3 version of Grimme’s dispersion model,^48^ while layer 2 was described by the AMBER force field.^49^ The optimized clusters were subsequently used in electronic structure calculations to determine one- and two-photon absorption spectra. These calculations were performed using TURBOMOLE program^50^ at the RI-CC2/aug-cc-pVDZ level of theory,^51^ and all solvent molecules were represented by point charges.

## 3 Results and Discussion

BF_2_-functionalized benzothiazoles with additional electron-donating functional methoxy groups were synthesized according to a protocol described elsewhere.^25^ These probes possess a single C–C bond between the benzothiazole core and phenylene group, which introduces rotational freedom—a feature resulting in the linear dependency of FQY on viscosity.^25^ However, the sensitivity of FQY to viscosity changes differs between dyes. It was proven that the FQY of **DA-** is mainly sensitive to viscosity, **-AD** is weakly sensitive to environmental factors (hydrogen-bonding network, polarity, and viscosity), and **DAD** is simultaneously sensitive to the hydrogen-bonding/polar environment and viscosity.^25^

**Figure 2:**
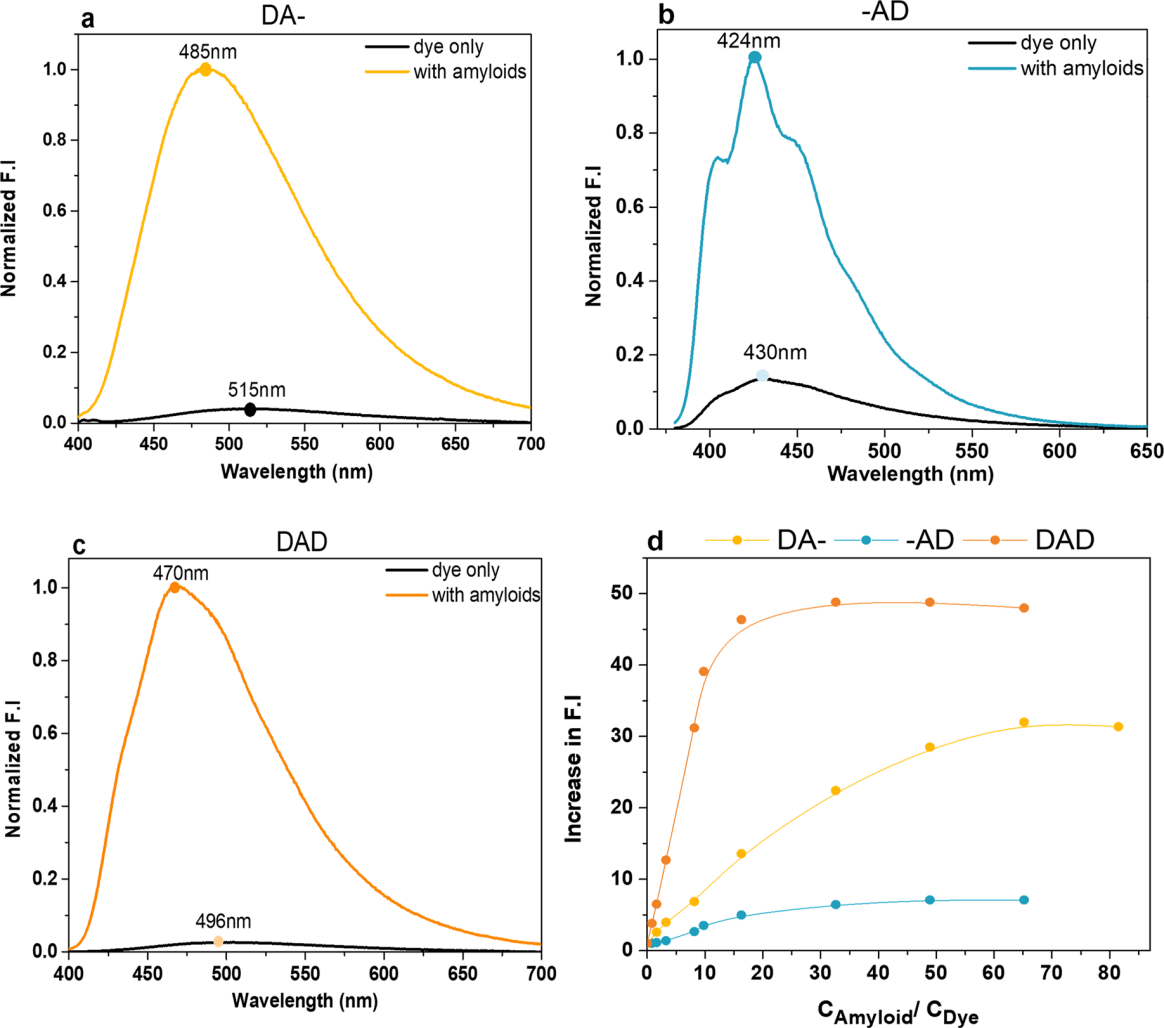
(a) Fluorescence intensity of **DA-** upon interaction with BI amyloids (122 μM). (b) Fluorescence intensity of **-AD** upon interaction with BI amyloids (122 μM). (c) Fluorescence intensity of **DAD** upon interaction with BI amyloids (82 μM). (d) Comparison of fluorescence increase with increasing concentration of BI amyloids.

The dyes present a low intensity of fluorescence in H_2_O/DMSO mixture, with FQY values equal to 1.3, 1.1, and 12.8% for **DAD**, **DA-**, and -**AD**, respectively. These values can be largely increased upon binding with amyloids, as shown in Figure 2a–c. We prepared bovine insulin (BI) amyloids and confirmed their presence by atomic force microscopy (AFM) (Figure S1a). Details about the preparation of samples are described in the Experimental Section. Values of the fluorescence intensity (FI) for amyloid solutions were compared before and after binding. As shown in Figure 2d, a strong increase in FI, from 7 for **-AD** dye to 49-fold for the **DAD** dye, was observed upon binding to amyloids. To determine whether the increase in FI is equally sensitive to protein monomers, the fluorescence of dye solutions with the monomeric form of bovine insulin was measured. A lack of intensity changes confirmed that the enhancement of FI is specific for the interaction with amyloid fibrils (Figure S6). As shown in Figure 2d, **DAD** achieves the highest fluorescence increase and **-AD** the lowest fluorescence increase for the same ratio between the amyloid and dye concentration. In order to evaluate the potential usage of dyes *in vivo*, the fluorescence response in a range of concentrations of bovine serum albumin (BSA) was determined (Figure S5). The smallest interactions seem to occur for the **-AD** dye, as with increasing BSA concentration, the fluorescence remains almost constant. For dyes **DAD** and **DA-**, we observed similar responses between 10 and 125 μM BSA. We clearly observed the interactions of BSA with these two dyes. It is worth mentioning that in the same instrumental settings, dyes with amyloids present higher intensities at lower concentrations compared to BSA. To conclude, there is a small chance of a background signal from the BSA–dye complex, especially for the **DAD** dye. However, the interactions suggest that it might be efficiently transported through the bloodstream.

For all dyes, the emission maximum was blue-shifted upon binding as compared to the solution without amyloids. One-photon optical properties such as the emission maximum wavelength (λ_em_ [nm]), absorption maximum wavelength (λ_abs_ [nm]), and average fluorescence lifetime (τ_avr_ [ns]) were also measured in H_2_O/DMSO mixtures as well as in solvents of low polarity (CHCl_3_) and high viscosity (glycerol), as presented in Table S1. For ease of comparison, we present the position of fluorescence emission for all dyes in used solvents in one spectrum (Figure S2). The smallest changes are observed for **-AD**. To examine the photostability of dyes, we measured the fluorescence intensity changes upon irradiation of 370 nm wavelength (Figure S3). The **DA-** and **-AD** intensities did not change, which indicates that they are highly photostable, while the **DAD** intensity decreased by around 10% after 1h of irradiation.

Amyloids possess a hydrophobic core and hydrophilic side groups exposed to the aqueous solution, and binding of dyes may occur in either of the locations. Dissolving dyes in CHCl_3_ is intended to show the optical properties of dyes in a hydrophobic medium, whereas glycerol is used to evaluate the influence of immobilization of dyes in the amyloid fibrils.

FQY and τ values were evaluated before and after amyloid binding in the same solvent. The highest FQY in the presence of amyloids was achieved for **-AD** (94%). This value is similar to the best FQY standards, such as Rhodamine 6G or fluorescein.^33^ The second highest value in the presence of amyloids was obtained for **DAD** and was equal to 75%. The least emissive is **DA-**, with an FQY equal to 27.5%. The decrease of FQY values in the series **-AD**, **DAD**, and **DA-** observed in amyloid solutions corresponds to the order found for solutions in chloroform (Table S1; note that the values are not the same).

First, we compared the FQY in the same solvents before and after binding to amyloids, and subsequently, we compared changes in FQY with the values in high-viscosity media (glycerol) as the dyes exhibited different sensitivity to viscosity. **DA-** showed a 25-fold increase in FQY (ϕ_rat_) upon binding to amyloids, with similar changes of FQY in amyloids and glycerol (Table 1). This suggests that the improved FQY comes mainly from the immobilization of the molecule upon binding to amyloids. The same behavior is observed for ThT, which belongs to the class of molecular rotor probes. In **-AD**, ϕ_*rat*_ upon adding amyloids was 7.4, which is higher than the change observed in glycerol in comparison to the much less viscous methanol. In any other solvent, this probe does not exhibit equally high FQY, even though it has similar values in CHCl_3_ and glycerol (Table S1).

**Table 1:** Selected Spectroscopic Data for the Dyes in Different Solvents

probe	solution	Φ [%]	Φ_rat_^a^	τ_avr_ [ns]
**DA-**	H_2_O/DMSO^b^	1.1		0.16
	amyloids^b^	27.5	25	1.93
	glycerol	11.4	27.1	1.08
**-AD**	H_2_O/DMSO^b^	12.8		0.42
	amyloids^b^	94.4	7.4	1.98
	glycerol	73.3	2.7	1.58
**DAD**	H_2_O/DMSO^b^	1.3		0.21
	amyloids^b^	75.2	57.9	2.45
	glycerol	28.4	11.8	1.15

a Φ_rat_ for the amyloid solution is Φ of the dye with amyloids divided by Φ of the dye in H_2_O/DMSO mixture. Φ_rat_ for glycerol is Φ of the dye in glycerol solution divided by Φ of the dye in methanol. Data for glycerol are taken from ref (26).

b Details on concentrations and v/v ratios between solvents for each dye are presented in the Experimental Section in the Supporting Information file.

The highest increase in FQY of the presented dyes (58-fold) upon the addition of amyloids was observed for **DAD** (Table 1). Its FQY with amyloids (75%) was almost two times higher than that for ThT with amyloids (42%),^34^ while the FQY was found to be lower for glycerol (viscous media) than for the solution of amyloids. This confirms our previous finding that the dye is sensitive to viscosity, and polarity/hydrogen bonding can translate into an interaction with amyloids.

**DAD** and **-AD** both have a methoxy group on the phenyl side. Some of the present authors have previously proved that such a location of the donor is crucial for achieving a high value of FQY.^25^ The methoxy moiety attached to the benzothiazole core present in **DAD** and **DA-** has already been used in ThT derivatives to increase the electron density on the benzothiazole core.^35,36^ ThX, one of the ThT derivatives, had an FQY 3.4 times higher than that of the parent ThT upon binding to α-synuclein and showed increased binding affinity. Another publication presented a comparison of a novel library of 12 ThT-inspired fluorescent probes for amyloid protein with the methoxy moiety attached to the benzothiazole core (with or without a positive charge in their structure).^36^ In general, charged molecules exhibit a higher FQY after binding to α-synuclein. Our studies show that the introduction of the BF_2_ moiety as the acceptor serves as a possible solution for achieving high FQY without using charged species, which is an interesting alternative in the design of probes for amyloid aggregates. Moreover, it should not be overlooked that the dyes carrying a quaternary/charged nitrogen atom would rather interact with polar groups within the protein, while BF_2_-carrying ones provide a chance to bind in hydrophobic parts.

Determination of τ and FQY values allowed us to calculate the radiative (*k*_r_) and nonradiative (*k*_nr_) decay constants and their changes upon dye binding to amyloids. Fluorescence lifetime values after binding were equal to 1.93, 1.98, and 2.45 for **DA-**, **-AD**, and **DAD**, respectively (Figure S3). In order to link the effect of binding to amyloids with changes in fluorescence, we compared the values of *k*_r_ and *k*_nr_ in CHCl_3_, glycerol, and H_2_O/DMSO solutions before and after amyloid addition for all probes.

**Figure 3:**
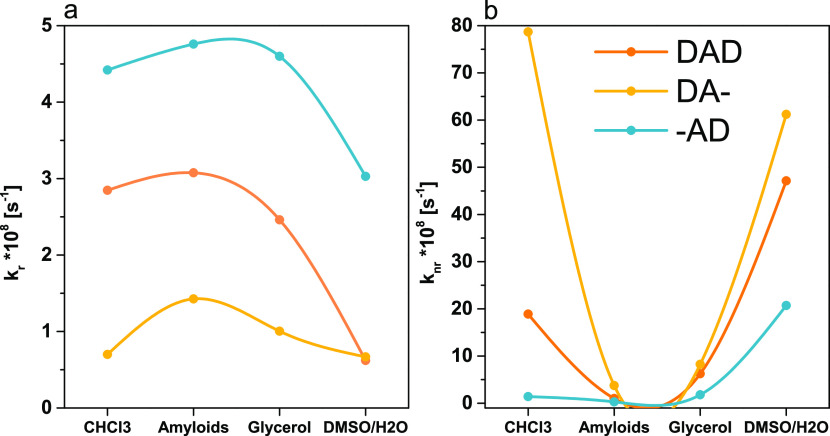
(a) Comparison of *k*_r_ values for dyes in CHCl_3_, amyloid solution, glycerol, DMSO/H_2_O. (b) Comparison of *k*_nr_ values for dyes in CHCl_3_, amyloid solution, glycerol, DMSO/H_2_O. Details of the calculations are given in the SI.

**DA-** presents a decrease in values of *k*_nr_ after amyloid binding (Figure 3b). There are similarities in *k*_nr_ in a high-viscosity medium and amyloids. There is additional confirmation that the restriction of the conformational freedom of a dye takes place, which translates into the suppression of the nonradiative processes. In the case of **DA-**, there is also an increase in *k*_r_ after binding to amyloids, with higher *k*_r_ in amyloids than in glycerol (Figure 3b).

**-AD** also presents suppression of nonradiative processes after amyloid binding. However, the dye presents similar values of *k*_nr_ in CHCl_3_, glycerol, and upon binding to amyloids. Thus, it is not possible to point out a single dominant environmental factor that contributes to the suppression of these processes. The FQY of **-AD** slightly changes with viscosity; thus, the immobilization of the dye contributes to a decrease of nonradiative decay pathways upon binding to amyloids. The contribution of *k*_r_ in an amyloid solution is the highest for this dye among all investigated dyes. The localization of the donor group may reduce the rotation capacity around the bond between the benzothiazole core and phenylene group,^37^ leading to an initial high FQY of a free dye. Then, upon dye binding to amyloids, further immobilization and hydrophobicity of grooves in amyloids, where the dye is located, allow us to achieve an FQY above 90%.

The **DAD** dye presents high suppression of nonradiative processes after amyloid binding. On a comparison, the *k*_nr_ values of different solvents were found to be the most similar to the *k*_nr_ value measured in glycerol. This proves that the immobilization of a molecule takes place, which results in a decrease of *k*_nr_. The acceleration of radiative pathways upon binding to amyloids is the highest for this system compared to other dyes (Table S3), which translates into the highest Δϕ upon binding to amyloids. As shown in Figure 3a, *k*_r_ has similar values in a hydrophobic solvent and amyloids; thus, the polarity may have a significant influence on the radiative decay pathways.

Based on a comparison between dyes, we link particular moieties with the changes of *k*_r_ and *k*_nr_. The localization of the donor on the part of a molecule exhibiting rotational freedom influences *k*_r_, while the donor attached to the benzothiazole core has a stronger influence on *k*_nr_. The highest FQY was obtained for **-AD** upon binding to amyloids, thus achieving a high FQY depending on electron density on the moiety exhibiting rotational freedom. The donor group attached to the benzothiazole core increases the sensitivity of FQY to the immobilization of dyes. **DAD** and **DA-** exhibit FQY sensitivity to viscosity and more “molecular rotor probe”-like behavior. For **DAD** and **-AD** dyes, *k*_r_ values in samples with amyloids correspond better with values in a hydrophobic environment than in glycerol. This suggests that dyes are located inside the hydrophobic amyloid structure, which is supported by the hydrophobic character of the dyes determined from log *P* values (see Table S4) and the mentioned earlier presence of fluorine atoms.

We also studied the optical properties of dyes in amyloids of other proteins. The fluorescence intensity of the compounds upon the addition of amyloids formed from hen egg white lysozyme (HEWL) was lower compared to that of BI amyloids (Figure S8), which we attribute to various amyloid environments, e.g., a higher content of arene groups in HEWL compared to BI. A detailed discussion regarding the probe performance upon binding to HEWL is presented in the SI. Overall, trends in intensity increase are the same for dyes upon binding to HEWL and BI amyloids. However, **-AD** in HEWL amyloids presented quenching, and no increment of FI upon binding was observed (Figure S9).

As **DAD** presented the highest increment of FQY upon binding and exhibited the best application potential, we also investigated interactions with various endogenous biomolecules (Figure S4). To summarize, we did not observe any changes in fluorescence upon interaction with to l-cysteine, l-methionine, glycine, glutathione, ascorbic acid, and H_2_O_2_.

**Figure 4:**
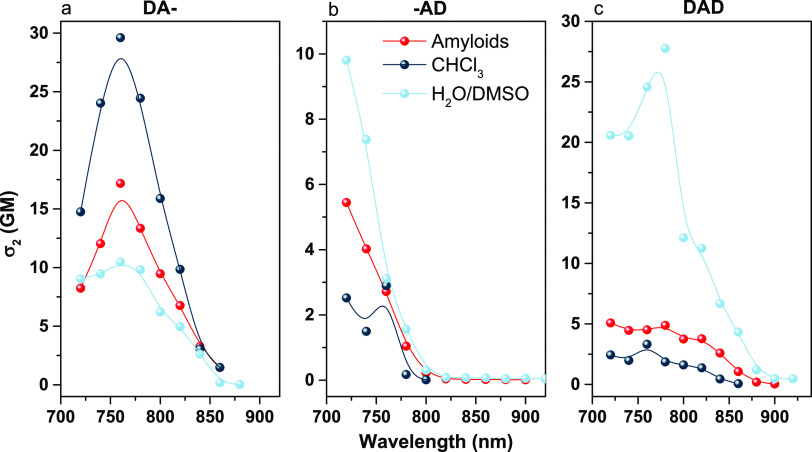
Two-photon absorption cross section for dyes in various environments: H_2_O/DMSO mixture (ratios for all dyes are presented in the Experimental Section), CHCl_3_, and amyloid solution (concentration presented in the Experimental Section). (a) **DA-** dye, (b) **-AD** dye, and (c) **DAD** dye. The uncertainty of the calculated values was ±15%.

Nonlinear optical properties of the analyzed chromophores were studied in terms of their multiphoton absorption and the corresponding fluorescence processes, with a femtosecond pulsed laser as an excitation source. First, the two-photon nature of absorption processes was confirmed with fluorescence response *vs* the input laser power (Figure S10). σ_2_ values of the dyes before and after amyloid binding were measured. The σ_2_ value in H_2_O/DMSO solution was found to be the highest for the **DAD** dye (28 GM at 780 nm) while the lowest was for **-AD** (∼10 GM at 720 nm; Figure 4). For **DA-**, σ_2_ increased upon binding to amyloids, whereas for **-AD**, a minor difference was observed, and for **DAD**, we noticed a decrease of the σ_2_ value. We also compared the two-photon absorption (2PA) spectra of all chromophores with their one-photon absorption (1PA) spectra (Figure S11). The 2PA spectra of free dyes overlap with the 1PA bands plotted at a double wavelength. On the other hand, probes with amyloids have slightly shifted 2PA bands compared to the doubled positions of the 1PA spectra (Figure S11). In order to assess the effect of polarity on σ_2_, we performed 2PA measurements in a more hydrophobic and less polar solvent—CHCl_3_. For **DAD** and **-AD** in CHCl_3_, σ_2_ decreased compared to that in the H_2_O/DMSO solution, while for **DA-**, σ_2_ was higher (Figure 4). The presented tendency of σ_2_ in CHCl_3_ correlates to changes upon adding amyloids. The dependence of σ_2_ on the solvent polarity is a well-known phenomenon.^38,39^ Binding pockets of amyloids are mostly hydrophobic,^40,41^ which translates to the observed correspondence between the two-photon performance of dyes in amyloids and CHCl_3_ solutions. However, values in these two solutions for all probes are not the same: for **DA-**, σ_2_ at 760 nm in CHCl_3_ is 29 GM, which is almost two times higher than that in the solution of amyloids. In complex biopolymers such as amyloids, additional factors influence 2PA, e.g., the local electric field.^31,42,43^ Previously presented differences between dyes in BI and HEWL amyloids indicate that interactions with amino acid residues present in fibrils significantly modulate the optical properties of dyes. Similarly, the modulation of nonlinear optical properties is expected.

The experimental investigation of two-photon properties of dyes in solutions was supported by quantum mechanics/molecular mechanics (QM/MM) simulations of dyes in CHCl_3_ and water (mimicking a water/DMSO mixture). Details of the simulations are provided in the Experimental Section, and a summary of the results is presented in Table S6. The results of simulations are in line with experimental findings and clearly indicate that σ_2_ of **DA-** in CHCl_3_ is much larger than those of **DAD** and **-AD**. Electronic structure calculations also indicate that a more polar environment (water *vs* CHCl_3_) increases the σ_2_ values of the studied dyes.

σ_eff_, which is a product of σ_2_ and FQY, is the quantity that is the most relevant for evaluating the potential of two-photon probes in imaging applications. Values of σ_eff_ increased upon binding for all investigated dyes (Figure S12) due to increasing FQY of these systems, with the value around 4–5 GM, for all dyes. However, the highest difference in the σ_eff_ value between the bound and unbound dyes was observed for **DA-** molecules, which presented the lowest FQY upon binding. In the case of sufficiently large values of σ_eff_, the increase of the two-photon excited fluorescence intensity upon incorporation into fibrils may be the fundamental factor determining the utility of a dye in bioimaging applications.

## 4 Conclusions

In conclusion, we presented a systematic study of the optical properties of three fluorophores with a structure based on ThT and an N,O-coordinated BF_2_ moiety, with a donor group located on both or one of the termini of a molecule (**DAD**, **DA-**, and -**AD**). We show that probes with an N,O-coordinated BF_2_ acceptor, while incorporated into amyloid fibrils, exhibit FQY values exceeding 70% even without the presence of a strong electron-donating moiety, like the frequently used *N*,*N*-dimethylamino group. The highest increase in fluorescence upon binding with amyloids was observed for the **DAD** dye, which exhibits an FQY sensitive to a range of environmental factors such as the hydrogen-bonding network, polarity, and viscosity. The results reveal that probes that are sensitive to more than one environmental factor might be the best choice in amyloid detection. Our data show that a crucial aspect in tuning the FQY is the donor location in the molecule. The best performance was obtained for a donor attached to the aromatic ring with a single C–C bond connected to the core with the acceptor. The methoxy group, as a hydrogen bond acceptor, enhances the interaction with amyloids in parallel with increasing FQY, while this group is attached to the benzothiazole ring.

The probes studied here presented various responses to HEWL and BI amyloids. It is beneficial as discrimination between various amyloid structures is important in bioimaging studies of protein aggregates. The probe **-AD** has an FQY that is the least sensitive to viscosity, and it presented the largest differences in fluorescence detected upon binding to HEWL *vs* BI. This might suggest that probes with limited sensitivity to viscosity perform better in discriminating between different types of amyloids, where different mechanisms influencing FQY play the main role.

We also investigated the two-photon properties of the dyes and proved the importance of measuring 2PA cross sections of dyes both in a solution and after amyloid binding. Measuring 2PA cross sections in solvents may show a trend but may not be sufficient for the prediction of two-photon properties of a dye upon binding to amyloids. The measurements of σ_2_ in organic solvents did not provide a reliable estimation of the difference in two-photon-excited fluorescence intensity between bound and unbound probes in water solutions, which is crucial for the observation of amyloid-bound dyes with no background fluorescence. Moreover, the evaluation of σ_2_ in amyloids gave us important information about the factors that contribute to the highest differences in two-photon properties between bound and unbound dyes. Such an approach can be used for a better comparison of various probes designed for amyloid imaging by two-photon microscopy.

## Supporting Information Available

AFM images of HEWL and BI amyloids; details of one-photon optical properties of dyes in different solvents; comparison of the fluorescence emissions with monomers of peptides and amyloids; fluorescence lifetime data; summary of radiative and nonradiative decay pathways; comparison of the fluorescence emission with HEWL amyloids and BI amyloids. partition coefficient (log *P*) of dyes; effective two-photon absorption cross sections; and results of computer simulations (PDF)

## References

[ref1] Iadanza M. G.; Jackson M. P.; Hewitt E. W.; Ranson N. A.; Radford S. E. A new era for understanding amyloid structures and disease. Nat. Rev. Mol. Cell Biol. 2018, 19 (12), 755–773. 10.1038/s41580-018-0060-8. 30237470 PMC7617691

[ref2] Tipping K. W.; van Oosten-Hawle P.; Hewitt E. W.; Radford S. E. Amyloid Fibres: Inert End-Stage Aggregates or Key Players in Disease?. Trends Biochem. Sci. 2015, 40 (12), 719–727. 10.1016/j.tibs.2015.10.002. 26541462 PMC7617725

[ref3] Yanagisawa D.; Taguchi H.; Ibrahim N. F.; Morikawa S.; Shiino A.; Inubushi T.; Hirao K.; Shirai N.; Sogabe T.; Tooyama I. Preferred Features of a Fluorine-19 MRI Probe for Amyloid Detection in the Brain. J. Alzheimer’s Dis. 2014, 39, 617–631. 10.3233/JAD-131025. 24246421

[ref4] Mathis C. A.; Mason N. S.; Lopresti B. J.; Klunk W. E. Development of Positron Emission Tomography β-Amyloid Plaque Imaging Agents. Semin. Nucl. Med. 2012, 42 (6), 423–432. 10.1053/j.semnuclmed.2012.07.001. 23026364 PMC3520098

[ref5] Gyasi Y. I.; Pang Y. P.; Li X. R.; Gu J. X.; Cheng X. J.; Liu J.; Xu T.; Liu Y. Biological applications of near infrared fluorescence dye probes in monitoring Alzheimer’s disease. Eur. J. Med. Chem. 2020, 187, 111982 10.1016/j.ejmech.2019.111982. 31877538

[ref6] Larson A. M. Multiphoton microscopy. Nat. Photonics 2011, 5 (1), 1 10.1038/nphoton.an.2010.2.

[ref7] Kobat D.; Durst M. E.; Nishimura N.; Wong A. W.; Schaffer C. B.; Xu C. Deep tissue multiphoton microscopy using longer wavelength excitation. Opt. Express 2009, 17 (16), 13354–13364. 10.1364/OE.17.013354. 19654740

[ref8] Chen C.; Liang Z.; Zhou B.; Li X.; Lui C.; Ip N. Y.; Qu J. Y. In Vivo Near-Infrared Two-Photon Imaging of Amyloid Plaques in Deep Brain of Alzheimer’s Disease Mouse Model. ACS Chem. Neurosci. 2018, 9 (12), 3128–3136. 10.1021/acschemneuro.8b00306. 30067906

[ref9] Korzhova V.; Marinković P.; Njavro J. R.; Goltstein P. M.; Sun F.; Tahirovic S.; Herms J.; Liebscher S. Long-term dynamics of aberrant neuronal activity in awake Alzheimer’s disease transgenic mice. Commun. Biol. 2021, 4 (1), 1368 10.1038/s42003-021-02884-7. 34876653 PMC8651654

[ref10] Mora A. K.; Murudkar S.; Alamelu A.; Singh P. K.; Chattopadhyay S.; Nath S. Benzothiazole-Based Neutral Ratiometric Fluorescence Sensor for Amyloid Fibrils. Chem. - Eur. J. 2016, 22 (46), 16505–16512. 10.1002/chem.201602981. 27727505

[ref11] Vus K.; Trusova V.; Gorbenko G.; Sood R.; Kinnunen P. Thioflavin T derivatives for the characterization of insulin and lysozyme amyloid fibrils in vitro: Fluorescence and quantum-chemical studies. J. Lumin. 2015, 159, 284–293. 10.1016/j.jlumin.2014.10.042.

[ref12] Sundaram G. S.; Garai K.; Rath N. P.; Yan P.; Cirrito J. R.; Cairns N. J.; Lee J. M.; Sharma V. Characterization of a brain permeant fluorescent molecule and visualization of Abeta parenchymal plaques, using real-time multiphoton imaging in transgenic mice. Org. Lett. 2014, 16 (14), 3640–3643. 10.1021/ol501264q. 25003699 PMC4372081

[ref13] Ono M.; Watanabe H.; Kimura H.; Saji H. BODIPY-based molecular probe for imaging of cerebral beta-amyloid plaques. ACS Chem. Neurosci. 2012, 3 (4), 319–324. 10.1021/cn3000058. 22860198 PMC3369805

[ref14] Tonali N.; Dodero V. I.; Kaffy J.; Hericks L.; Ongeri S.; Sewald N. Real-Time BODIPY-Binding Assay To Screen Inhibitors of the Early Oligomerization Process of Abeta1–42 Peptide. ChemBioChem 2020, 21 (8), 1129–1135. 10.1002/cbic.201900652. 31702868 PMC7217026

[ref15] Sen A.; Mora A. K.; Koli M.; Mula S.; Kundu S.; Nath S. Sensing lysozyme fibrils by salicylaldimine substituted BODIPY dyes - A correlation with molecular structure. Int. Biol. Macromol. 2022, 220, 901–909. 10.1016/j.ijbiomac.2022.08.112. 35998856

[ref16] Ran C.; Xu X.; Raymond S. B.; Ferrara B. J.; Neal K.; Bacskai B. J.; Medarova Z.; Moore A. Design, Synthesis, and Testing of Difluoroboron-Derivatized Curcumins as Near-Infrared Probes for in Vivo Detection of Amyloid-β Deposits. J. Am. Chem. Soc. 2009, 131 (42), 15257–15261. 10.1021/ja9047043. 19807070 PMC2784241

[ref17] Liu K.; Guo T. L.; Chojnacki J.; Lee H. G.; Wang X.; Siedlak S. L.; Rao W.; Zhu X.; Zhang S. Bivalent ligand containing curcumin and cholesterol as fluorescence probe for Abeta plaques in Alzheimer’s disease. ACS Chem. Neurosci. 2012, 3 (2), 141–146. 10.1021/cn200122j. 22685625 PMC3367438

[ref18] Kelley M.; Sant’Anna R.; Fernandes L.; Palhano F. L. Pentameric Thiophene as a Probe to Monitor EGCG’s Remodeling Activity of Mature Amyloid Fibrils: Overcoming Signal Artifacts of Thioflavin T. ACS Omega 2021, 6 (12), 8700–8705. 10.1021/acsomega.1c00680. 33817533 PMC8015118

[ref19] Calvo-Rodriguez M.; Hou S. S.; Snyder A. C.; Dujardin S.; Shirani H.; Nilsson K. P. R.; Bacskai B. J. In vivo detection of tau fibrils and amyloid beta aggregates with luminescent conjugated oligothiophenes and multiphoton microscopy. Acta Neuropathol. Commun. 2019, 7 (1), 171 10.1186/s40478-019-0832-1. 31703739 PMC6839235

[ref20] Liu H.; Kim C.; Haldiman T.; Sigurdson C. J.; Nystrom S.; Nilsson K. P. R.; Cohen M. L.; Wisniewski T.; Hammarstrom P.; Safar J. G. Distinct conformers of amyloid beta accumulate in the neocortex of patients with rapidly progressive Alzheimer’s disease. J. Biol. Chem. 2021, 297 (5), 101267 10.1016/j.jbc.2021.101267. 34599965 PMC8531671

[ref21] Stsiapura V. I.; Maskevich A. A.; Kuzmitsky V. A.; Uversky V. N.; Kuznetsova I. M.; Turoverov K. K. Thioflavin T as a Molecular Rotor: Fluorescent Properties of Thioflavin T in Solvents with Different Viscosity. J. Phys. Chem. B 2008, 112, 15893 10.1021/jp805822c. 19367903

[ref22] Kim D.; Moon H.; Baik S. H.; Singha S.; Jun Y. W.; Wang T.; Kim K. H.; Park B. S.; Jung J.; Mook-Jung I.; Ahn K. H. Two-Photon Absorbing Dyes with Minimal Autofluorescence in Tissue Imaging: Application to in Vivo Imaging of Amyloid-β Plaques with a Negligible Background Signal. J. Am. Chem. Soc. 2015, 137 (21), 6781–6789. 10.1021/jacs.5b03548. 25951499

[ref23] Cao K.; Farahi M.; Dakanali M.; Chang W. M.; Sigurdson C. J.; Theodorakis E. A.; Yang J. Aminonaphthalene 2-Cyanoacrylate (ANCA) Probes Fluorescently Discriminate between Amyloid-β and Prion Plaques in Brain. J. Am. Chem. Soc. 2012, 134 (42), 17338–17341. 10.1021/ja3063698. 22866977 PMC3480552

[ref24] Wang Y.-L.; Fan C.; Xin B.; Zhang J.-P.; Luo T.; Chen Z.-Q.; Zhou Q.-Y.; Yu Q.; Li X.-N.; Huang Z.-L.; Li C.; Zhu M.-Q.; Tang B. Z. AIE-based super-resolution imaging probes for -amyloid plaques in mouse brains. Mater. Chem. Front. 2018, 2 (8), 1554–1562. 10.1039/C8QM00209F.

[ref25] Rybczyński P.; Bousquet M. H. E.; Kaczmarek-Kȩdziera A.; Jȩdrzejewska B.; Jacquemin D.; Ośmialowski B. Controlling the fluorescence quantum yields of benzothiazole-difluoroborates by optimal substitution. Chem. Sci. 2022, 13 (45), 13347–13360. 10.1039/D2SC05044G. 36507166 PMC9682896

[ref26] Loudet A.; Burgess K. BODIPY Dyes and Their Derivatives: Syntheses and Spectroscopic Properties. Chem. Rev. 2007, 107 (11), 4891–4932. 10.1021/cr078381n. 17924696

[ref27] Liu M.; Ma S.; She M.; Chen J.; Wang Z.; Liu P.; Zhang S.; Li J. Structural modification of BODIPY: Improve its applicability. Chin. Chem. Lett. 2019, 30 (10), 1815–1824. 10.1016/j.cclet.2019.08.028.

[ref28] Bednarska J.; Zaleśny R.; Wielgus M.; Jędrzejewska B.; Puttreddy R.; Rissanen K.; Bartkowiak W.; Ågren H.; Ośmiałowski B. Two-photon absorption of BF2-carrying compounds: insights from theory and experiment. Phys. Chem. Chem. Phys. 2017, 19 (8), 5705–5708. 10.1039/C7CP00063D. 28177027

[ref29] Chen Y.; Ouyang Q.; Li Y.; Zeng Q.; Dai B.; Liang Y.; Chen B.; Tan H.; Cui M. Evaluation of N, O-Benzamide difluoroboron derivatives as near-infrared fluorescent probes to detect beta-amyloid and tau tangles. Eur. J. Med. Chem. 2022, 227, 113968 10.1016/j.ejmech.2021.113968. 34752954

[ref30] Chen Y.; Yuan C.; Xie T.; Li Y.; Dai B.; Zhou K.; Liang Y.; Dai J.; Tan H.; Cui M. N,O-Benzamide difluoroboron complexes as near-infrared probes for the detection of beta-amyloid and tau fibrils. Chem. Commun. 2020, 56 (53), 7269–7272. 10.1039/D0CC02820G. 32475993

[ref31] Drobizhev M.; Makarov N. S.; Tillo S. E.; Hughes T. E.; Rebane A. Two-photon absorption properties of fluorescent proteins. Nat. Methods 2011, 8 (5), 393–399. 10.1038/nmeth.1596. 21527931 PMC4772972

[ref32] Doan P. H.; Pitter D. R.; Kocher A.; Wilson J. N.; Goodson T. 3rd Two-Photon Spectroscopy as a New Sensitive Method for Determining the DNA Binding Mode of Fluorescent Nuclear Dyes. J. Am. Chem. Soc. 2015, 137 (29), 9198–9201. 10.1021/jacs.5b02674. 26121006

[ref33] Magde D.; Wong R.; Seybold P. G. Fluorescence Quantum Yields and Their Relation to Lifetimes of Rhodamine 6G and Fluorescein in Nine Solvents: Improved Absolute Standards for Quantum Yields. Photochem. Photobiol. 2002, 75 (4), 327–334. 10.1562/0031-8655(2002)0750327FQYATR2.0.CO2. 12003120

[ref34] Sulatskaya A. I.; Maskevich A. A.; Kuznetsova I. M.; Uversky V. N.; Turoverov K. K. Fluorescence quantum yield of thioflavin T in rigid isotropic solution and incorporated into the amyloid fibrils. PLoS One 2010, 5 (10), e15385 10.1371/journal.pone.0015385. 21048945 PMC2966444

[ref35] Needham L.-M.; Weber J.; Varela J. A.; Fyfe J. W. B.; Do D. T.; Xu C. K.; Tutton L.; Cliffe R.; Keenlyside B.; Klenerman D.; Dobson C. M.; Hunter C. A.; Müller K. H.; O’Holleran K.; Bohndiek S. E.; Snaddon T. N.; Lee S. F. ThX – a next-generation probe for the early detection of amyloid aggregates. Chem. Sci. 2020, 11 (18), 4578–4583. 10.1039/C9SC04730A. 34122915 PMC8159457

[ref36] Needham L.-M.; Weber J.; Pearson C. M.; Do D. T.; Gorka F.; Lyu G.; Bohndiek S. E.; Snaddon T. N.; Lee S. F. A Comparative Photophysical Study of Structural Modifications of Thioflavin T-Inspired Fluorophores. J. Phys. Chem. Lett. 2020, 11 (19), 8406–8416. 10.1021/acs.jpclett.0c01549. 32924494 PMC8741274

[ref37] Jędrzejewska B.; Skotnicka A.; Laurent A. D.; Pietrzak M.; Jacquemin D.; Ośmiałowski B. Influence of the Nature of the Amino Group in Highly Fluorescent Difluoroborates Exhibiting Intramolecular Charge Transfer. J. Org. Chem. 2018, 83, 7779–7788. 10.1021/acs.joc.8b00664. 29931971

[ref38] Wielgus M.; Michalska J.; Samoć M.; Bartkowiak W. Two-photon solvatochromism III: Experimental study of the solvent effects on two-photon absorption spectrum of p-nitroaniline. Dyes Pigm. 2015, 113, 426–434. 10.1016/j.dyepig.2014.09.009.

[ref39] Nag A.; Goswami D. Solvent effect on two-photon absorption and fluorescence of rhodamine dyes. J. Photochem. Photobiol., A 2009, 206 (2), 188–197. 10.1016/j.jphotochem.2009.06.007. PMC2778798 19946642

[ref40] Cao Q.; Boyer D. R.; Sawaya M. R.; Abskharon R.; Saelices L.; Nguyen B. A.; Lu J.; Murray K. A.; Kandeel F.; Eisenberg D. S. Cryo-EM structures of hIAPP fibrils seeded by patient-extracted fibrils reveal new polymorphs and conserved fibril cores. Nat. Struct. Mol. Biol. 2021, 28 (9), 724–730. 10.1038/s41594-021-00646-x. 34518699 PMC10396428

[ref41] Li B.; Ge P.; Murray K. A.; Sheth P.; Zhang M.; Nair G.; Sawaya M. R.; Shin W. S.; Boyer D. R.; Ye S.; Eisenberg D. S.; Zhou Z. H.; Jiang L. Cryo-EM of full-length α-synuclein reveals fibril polymorphs with a common structural kernel. Nat. Commun. 2018, 9 (1), 3609 10.1038/s41467-018-05971-2. 30190461 PMC6127345

[ref42] Drobizhev M.; Tillo S.; Makarov N. S.; Hughes T. E.; Rebane A. Color Hues in Red Fluorescent Proteins Are Due to Internal Quadratic Stark Effect. J. Phys. Chem. B 2009, 113 (39), 12860–12864. 10.1021/jp907085p. 19775174 PMC2893592

[ref43] Bairu S.; Ramakrishna G. Two-photon absorption properties of chromophores in micelles: electrostatic interactions. J. Phys. Chem. B 2013, 117 (36), 10484–10491. 10.1021/jp405416d. 23952702

[ref44] Grelich-Mucha M.; Lipok M.; Różycka M.; Samoć M.; Olesiak-Bańska J. One- and Two-Photon Excited Autofluorescence of Lysozyme Amyloids. J. Phys. Chem. Lett. 2022, 13 (21), 4673–4681. 10.1021/acs.jpclett.2c00570. 35605187 PMC9169060

[ref45] Makarov N. S.; Drobizhev M.; Rebane A. Two-photon absorption standards in the 550–1600 nm excitation wavelength range. Opt. Express 2008, 16 (6), 4029–4047. 10.1364/OE.16.004029. 18542501

[ref46] Chung L. W.; Sameera W. M. C.; Ramozzi R.; Page A. J.; Hatanaka M.; Petrova G. P.; Harris T. V.; Li X.; Ke Z.; Liu F.; Li H.-B.; Ding L.; Morokuma K. The ONIOM Method and Its Applications. Chem. Rev. 2015, 115 (12), 5678–5796. 10.1021/cr5004419. 25853797

[ref47] Frisch M. J.; Trucks G. W.; Schlegel H. B.; Scuseria G. E.; Robb M. A.; Cheeseman J. R.; Scalmani G.; Barone V.; Petersson G. A.; Nakatsuji H.; Li X.; Caricato M.; Marenich A. V.; Bloino J.; Janesko B. G.; Gomperts R.; Mennucci B.; Hratchian H. P.; Ortiz J. V.; Izmaylov A. F.; Sonnenberg J. L.; Williams-Young D.; Ding F.; Lipparini F.; Egidi F.; Goings J.; Peng B.; Petrone A.; Henderson T.; Ranasinghe D.; Zakrzewski V. G.; Gao J.; Rega N.; Zheng G.; Liang W.; Hada M.; Ehara M.; Toyota K.; Fukuda R.; Hasegawa J.; Ishida M.; Nakajima T.; Honda Y.; Kitao O.; Nakai H.; Vreven T.; Throssell K.; Montgomery J. A. Jr.; Peralta J. E.; Ogliaro F.; Bearpark M. J.; Heyd J. J.; Brothers E. N.; Kudin K. N.; Staroverov V. N.; Keith T. A.; Kobayashi R.; Normand J.; Raghavachari K.; Rendell A. P.; Burant J. C.; Iyengar S. S.; Tomasi J.; Cossi M.; Millam J. M.; Klene M.; Adamo C.; Cammi R.; Ochterski J. W.; Martin R. L.; Morokuma K.; Farkas O.; Foresman J. B.; Fox D. J. Gaussian 16, revision C.01; Gaussian, Inc.: Wallingford CT, 2016.

[ref48] Grimme S.; Antony J.; Ehrlich S.; Krieg H. A consistent and accurate ab initio parameterization of density functional dispersion correction (DFT-D) for the 94 elements H-Pu. J. Chem. Phys. 2010, 132, 154104 10.1063/1.3382344. 20423165

[ref49] Cornell W. D.; Cieplak P.; Bayly C. I.; Gould I. R.; Merz K. M. Jr.; Ferguson D. M.; Spellmeyer D. C.; Fox T.; Caldwell J. W.; Kollman P. A. A second generation force-field for the simulation of proteins, nucleic-acids, and organic-molecules. J. Am. Chem. Soc. 1995, 117, 5179–5197. 10.1021/ja00124a002.

[ref50] TURBOMOLE V7.2 2017, a development of University of Karlsruhe and Forschungszentrum Karlsruhe GmbH: 1989–2007, TURBOMOLE GmbH, since 2007; available from http://www.turbomole.com.

[ref51] Friese D. H.; Hättig C.; Ruud K. Calculation of two-photon absorption strengths with the approximate coupled cluster singles and doubles model CC2 using the resolution-of-identity approximation. Phys. Chem. Chem. Phys. 2012, 14, 1175–1184. 10.1039/C1CP23045J. 22130199

