# Supplementary material for: BF2-Functionalized Benzothiazole Amyloid Markers: Effect of Donor Substituents on One- and Two-Photon Properties
Source: ACS Appl Bio Mater. 2023 Dec 7;6(12):5676–84. doi: 10.1021/acsabm.3c00815 (PMC10731634; doi:10.1021/acsabm.3c00815)
Supplement: Supplementary file 1 — mt3c00815_si_001.pdf [file mt3c00815_si_001.pdf]

Supporting Information

BF_2-functionalized benzothiazole amyloid markers: the effect of donor substituents on one- and two-photon properties

Agata Hajda[1], Manuela Grelich-Mucha[1], Patryk Rybczyński[2], Borys Ośmiałowski[2], Robert Zaleśny[1], Joanna Olesiak-Bańska[1*]

1.Faculty of Chemistry, Wroclaw University of Science and Technology, Wybrzeże Wyspiańskiego 27, 50-370 Wroclaw, Poland

2. Faculty of Chemistry, Nicolaus Copernicus University, Gagarina Street 7, Toruń, PL-87-100, Poland

a)

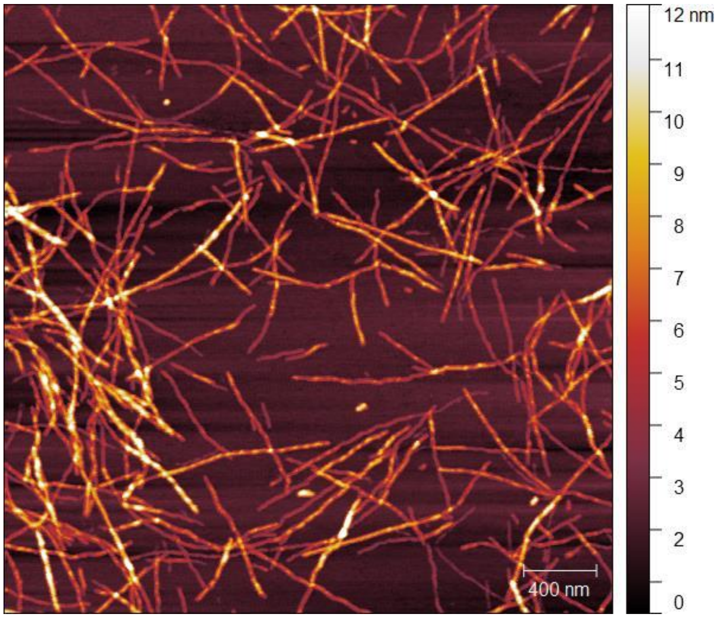

b)

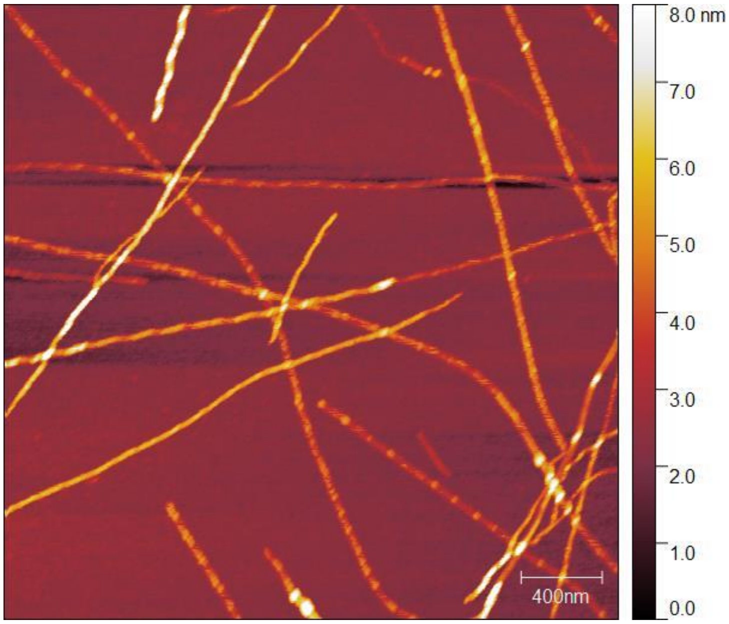

Figure S1. (a) Amyloids of BI deposited on mica (b) Amyloids of HEWL deposited on mica.

Table S1. One-photon optical properties of dyes in different solvents.

Probe	Solvent	Φ [%]	λ_{em}[nm]	λ_{abs} [nm]
**DA-**	H_2O/DMSO	1.1	515	361.0
	$CHCl_3$	0.9	458.5	361.0
	DMSO	-	512.0	365.0
	Glycerol	11.4	491.5	-
	Amyloids	27.5	485	364
**-AD**	H_2O/DMSO	12.7	430.0	364.0
	$CHCl_3$	75.6	419.5	364.0
	DMSO	-	428.0	366.0
	Glycerol	73.3	426.5	-
	Amyloids	94.4	424	366
**DAD**	H_2O/DMSO	1.3	502.5	373.0
	$CHCl_3$	13.1	454.5	368.0
	DMSO	-	501.0	375.0
	Glycerol	28.3	482.0	-
	Amyloids	75.2	470	380

For $CHCl_3$ and glycerol data taken from: Rybczynski, P.; Bousquet, M. H. E.; Kaczmarek-Kedziera, A.; Jedrzejewska, B.; Jacquemin, D.; Osmialowski, B., Controlling the fluorescence quantum yields of benzothiazole-difluoroborates by optimal substitution. *Chem Sci* **2022,** *13* (45), 13347-13360.

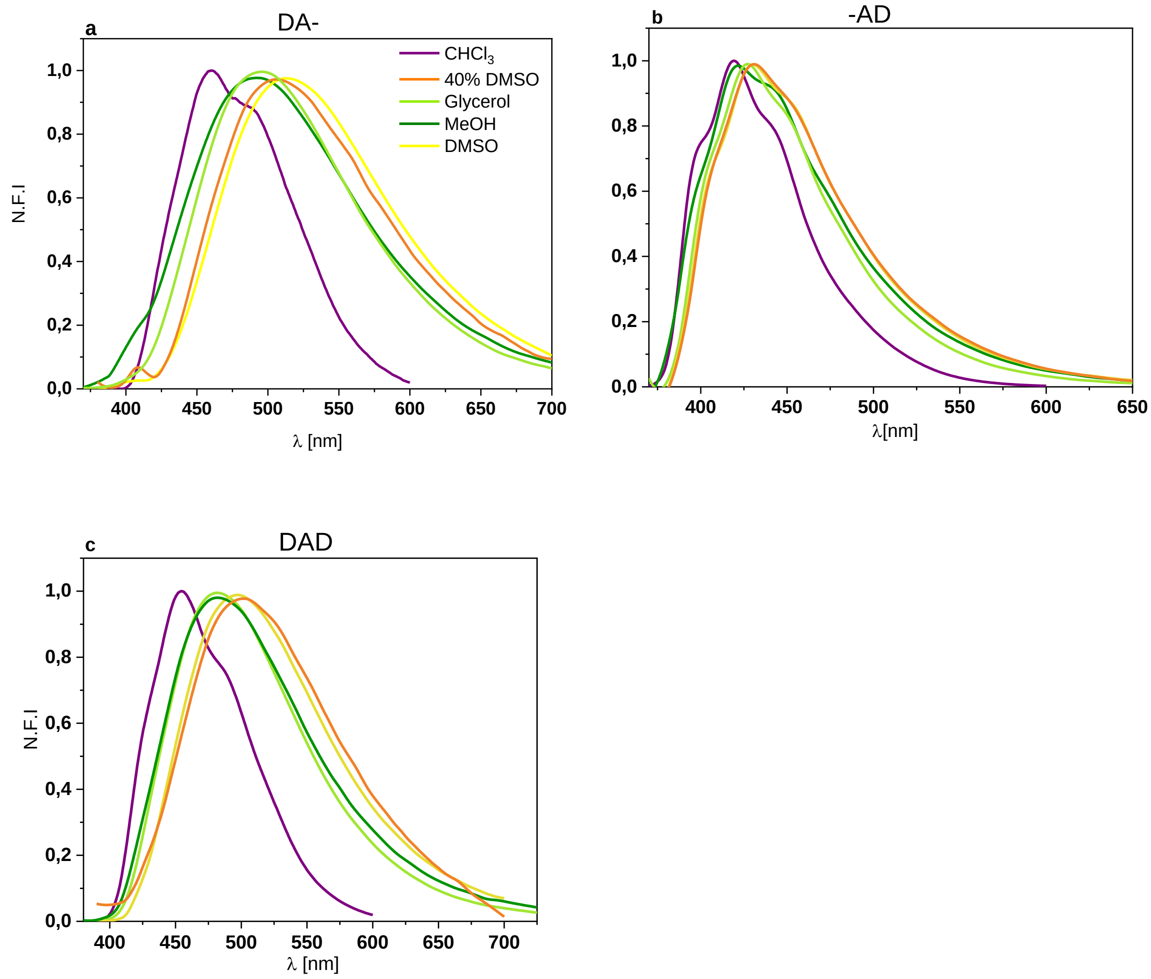

Figure S2. (a) DA- normalized fluorescence emission in different solvents (b) -AD normalized fluorescence emission in different solvents (c) DAD normalized fluorescence emission in different solvents.

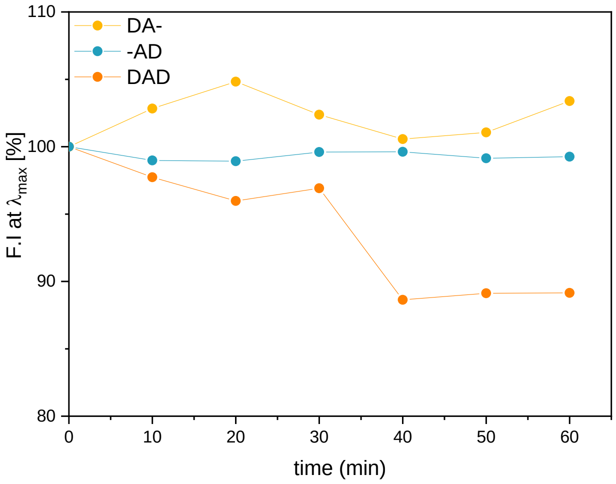

Figure S3. Photostability examination under irradiation using Xenon Lamp at 370nm. Fluorescence intesity at maximum emission wavelength before irradiation and during irradiation was monitored. Changes in intesity were recalculated to procentage for better visualization of data.

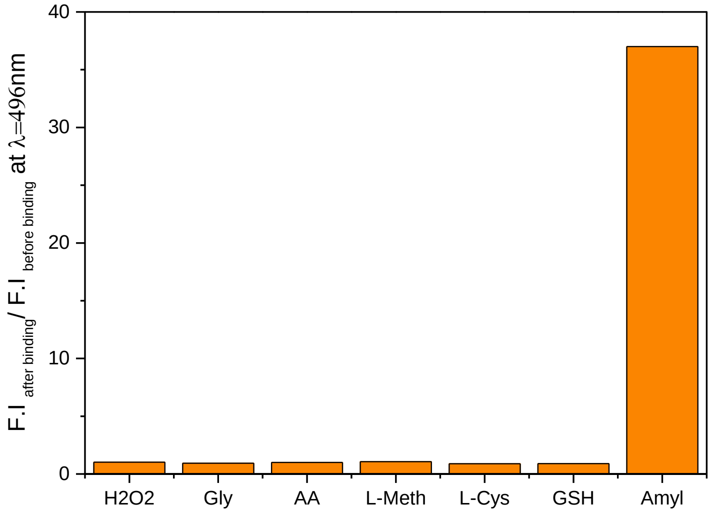

Figure S4. Fluorescence increasement of 2.5uM dye DAD upon mixing with various endogenous biomolecules – 100uM H_2O_2, 5mM Glycine (Gly), 10mM Ascorbic Acid(AA), 5mM L-Methionine(L-Meth), 5mM L-Cysteine (L-Cys), 1mM L-Gluthiahione (GSH) and comapred to increasment upon binding to 82uM amyloids from bovine insulin.

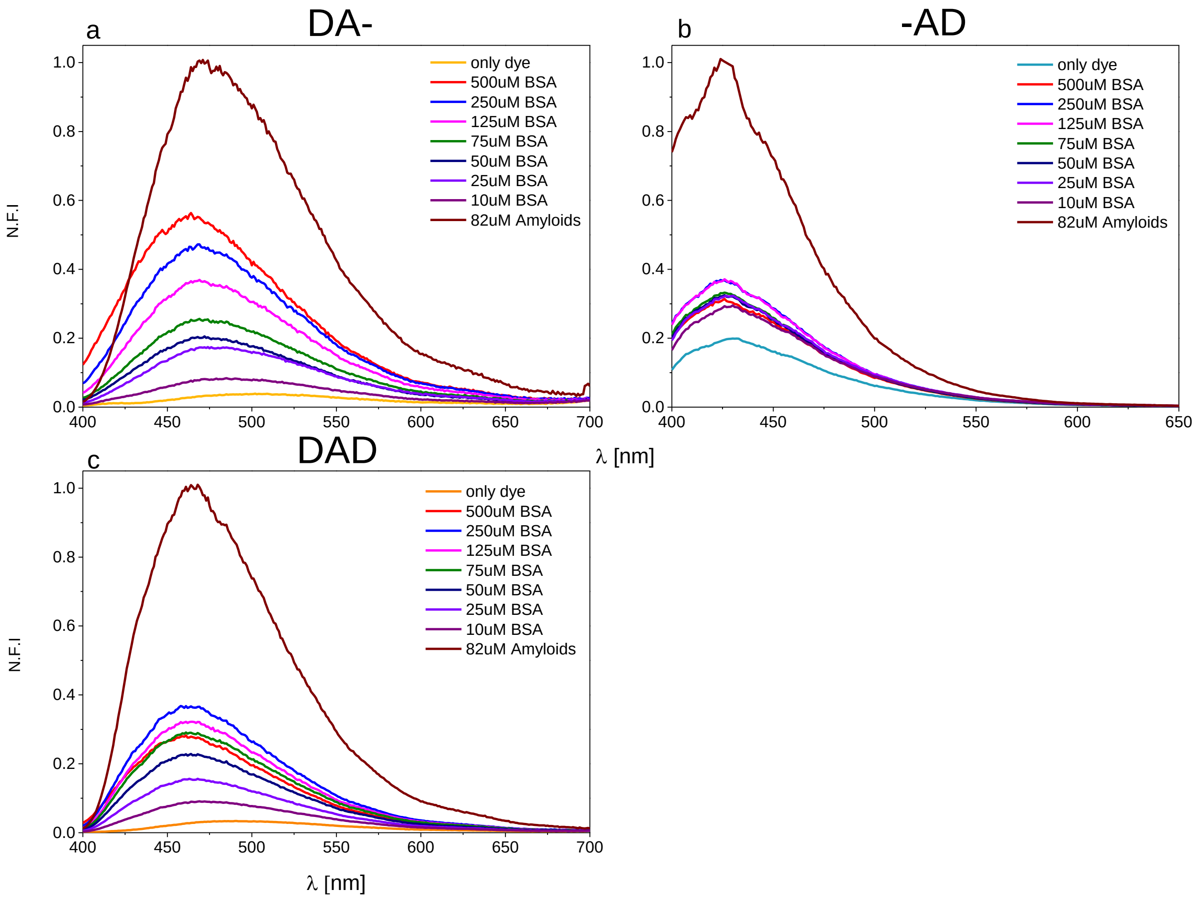

Figure S5 . Comparison of fluorescence intensity changes upon binding of dyes (c=2.5uM) to various concentrations of BSA. Comparison of fluorescence emission was taken before and after binding for maximum emission wavelength upon binding to BSA and to enhancement upon binding 82uM bovine amyloids.

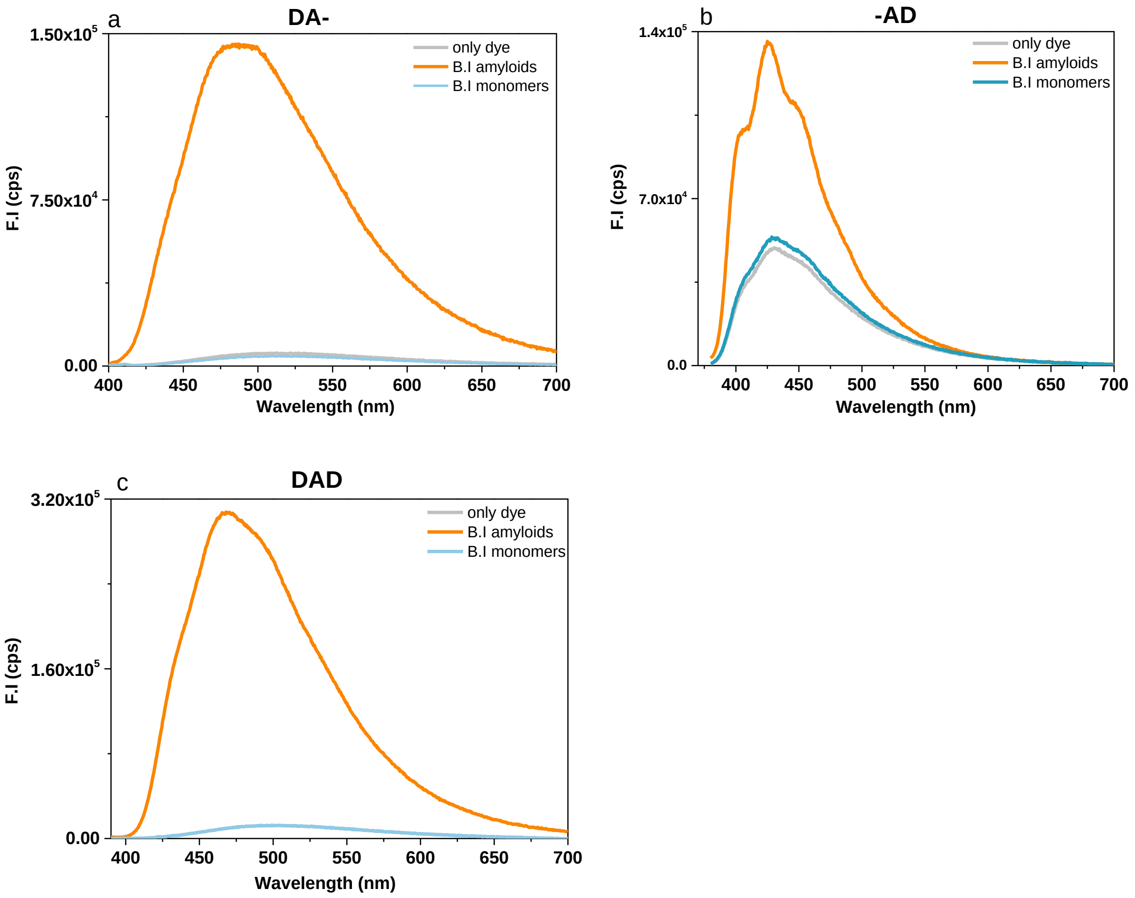

Figure S6. (a) comparison of F.I for dye (DA-) upon adding amyloids and monomers of bovine insulin (20uM); (b) comparison of F.I for dye (-AD) upon adding amyloids and monomers of bovine insulin (20uM); (c) comparison of F.I for dye (DAD) upon adding amyloids and monomers of bovine insulin (20uM).

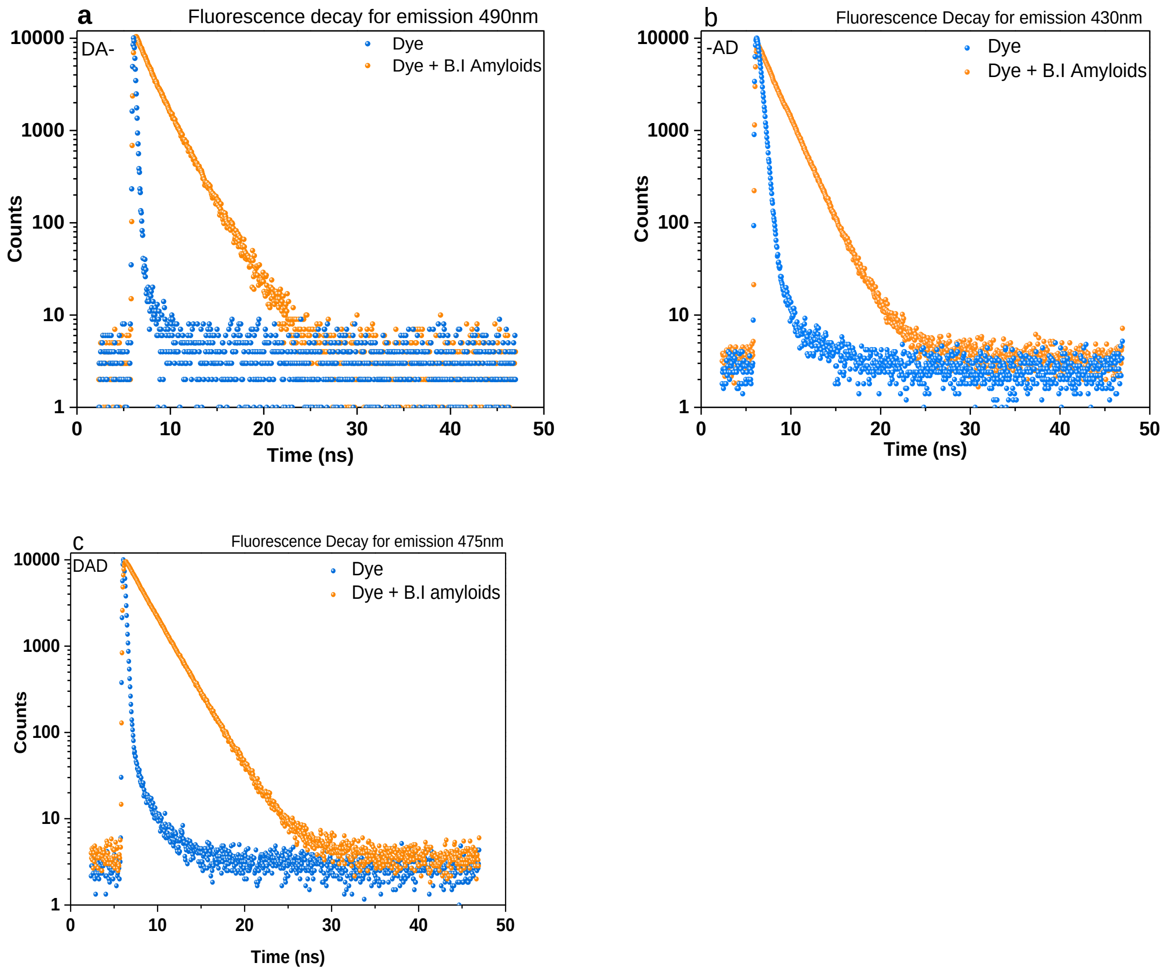

Figure S7. Fluorescence lifetime fitting for dyes with maximal amyloid F.I increment (a) DA- (b) -AD (c) DAD dye.

Tabel S2. Fluorescence lifetime details for water/dmso mixture and amyloid solution (maximal F.I enhancement concentration).

Probe	**solution**	τ_1[ns]	a%	τ_2[ns]	b%	τ_{avr}[ns]
DA-	40%DMSO	0.157	99.7	1.013	0.3	0.16
	Amyloid	1.509	56%	2.489	44%	1.93
-AD	40%DMSO	0.419	100%			0.42
	Amyloid	1.982	100%	-	-	1.98
DAD	40%DMSO	0.201	99,7%	1.827	0.3%	0.21
	Amyloid	2.445	100%	-	-	2.45

Table S3. Summary of radiative and non-radiative decay pathways

DYE	Solution	Φ [%]	τ_{avr}[ns]	k_{nr} (*10^8) [s^{-1}]	k_r (*10^8) [s^{-1}]
**DA-**	H_2O/DMSO	1.1	0.16	61.2	0.7
	Amyloids	27.5	1.93	3.7	1.4
	MeOH[1]	0.4	0.12	84.5	0.4
	Glycerol[1]	11.4	1.08	8.2	1.1
	$CHCl_3$[1]	0.9	0.13	78.7	0.7
**-AD**	H_2O/DMSO	12.7	0.42	20.7	3.0
	Amyloids	94.4	1.98	0.3	4.8
	MeOH[1]	26.5	0.68	10.8	3.9
	Glycerol[1]	73.3	1.58	1.7	4.6
	$CHCl_3$[1]	75.6	1.71	1.4	4.4
**DAD**	H_2O/DMSO	1.33	0.21	47.1	0.7
	Amyloids	75.3	2.45	1.0	3.2
	MeOH[1]	2.4	0.14	72.3	1.8
	Glycerol[1]	28.4	1.15	6.2	2.5
	$CHCl_3$[1]	13.1	0.46	18.9	2.85

[1] Data taken from: Rybczynski, P.; Bousquet, M. H. E.; Kaczmarek-Kedziera, A.; Jedrzejewska, B.; Jacquemin, D.; Osmialowski, B., Controlling the fluorescence quantum yields of benzothiazole-difluoroborates by optimal substitution. *Chem. Sci.* 2022, 13 (45), 13347-13360.

Table S4. Partition Coefficient (LogP) - Calculated using the online ALOGPS 2.1 program

DYE	logP
DA-	4.88
-AD	4.93
DAD	4.94

HEWL amyloid experiments

Fluorescence emissions of compounds upon addition of amyloids formed from hen egg white lysozyme (HEWL) was evaluated (Figure S4). Presence of HEWL amyloids was confirmed by AFM imaging (Figure S1b). All probes with HEWL amyloids presented smaller fluorescence intensity compared to BI amyloids. F.I for **-AD** with HEWL was smaller than for the dye without amyloids, which suggests fluorescence quenching mechanism upon binding. Increasing concentration of amyloids caused further quenching (Figure S5). Amyloids, besides their structural similarities, differ according to the amino acids content. Unique microenvironment, coming from amino acids residues, is used to distinguish amyloids by fluorescent probes.[2] It is known phenomenon for ThT[3] and Nile Red[4] that binding to HEWL amyloids results in smaller emission enhancement compared to BI amyloids. Since ThT is a charged molecule, electrostatic interactions play an important role influencing binding to amyloids. The molecules studied in this Letter are neutral; therefore, the electrostatic interactions do not prevail over other interaction components and we thus do not include them in our considerations. HEWL amyloids have higher hydrophobicity and contain more aromatic moieties within the groove than bovine insulin amyloids.[5] The arene groups play important role, since HEWL have six Tryptophan (Trp) residues in the sequence whereas BI have none. Quenching of dyes luminescence caused by Trp in well-known phenomenon.[6] The π-π interaction creating various microenvironment of studied amyloids grooves, including Trp presence, may be responsible for different optical properties of probes. This finding also supports our previous divagation about interaction of dyes with amino acids residues. **DAD** and **DA-** both have higher dependence of fluorescence on viscosity than **-AD** and for these dyes HEWL amyloids induced the increase in F.I upon binding.

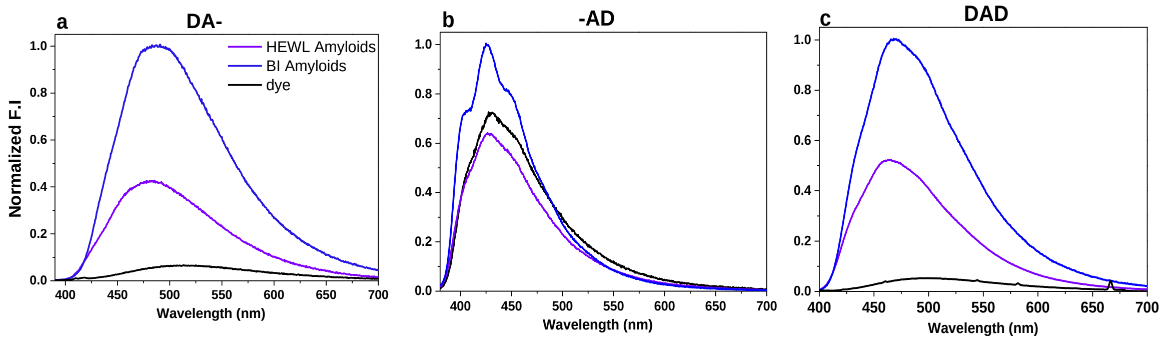

Figure S8. Comparison of Normalized Fluorescence Intensity (N.F.I) of dyes upon binding to amyloids of HEWL and BI. Both had concentration 20uM. Normalization was done using the highest Fluorescence intensity as 1.

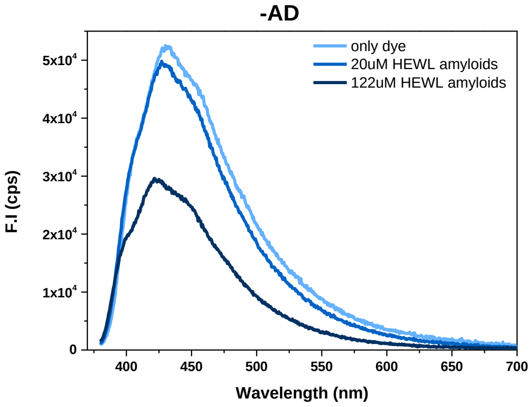

Figure S9. Fluorescence emission of -AD (2.5uM) upon binding to HEWL amyloids.

Table S5. Summarizing table of emission properties of dyes with 20uM of HEWL amyloids.

DYE	λ_{em} [nm]	Fold
DA-	482	8
-AD	427	decrease
DAD	462	15

Fold – the increase of fluorescence intensity upon binding with amyloids

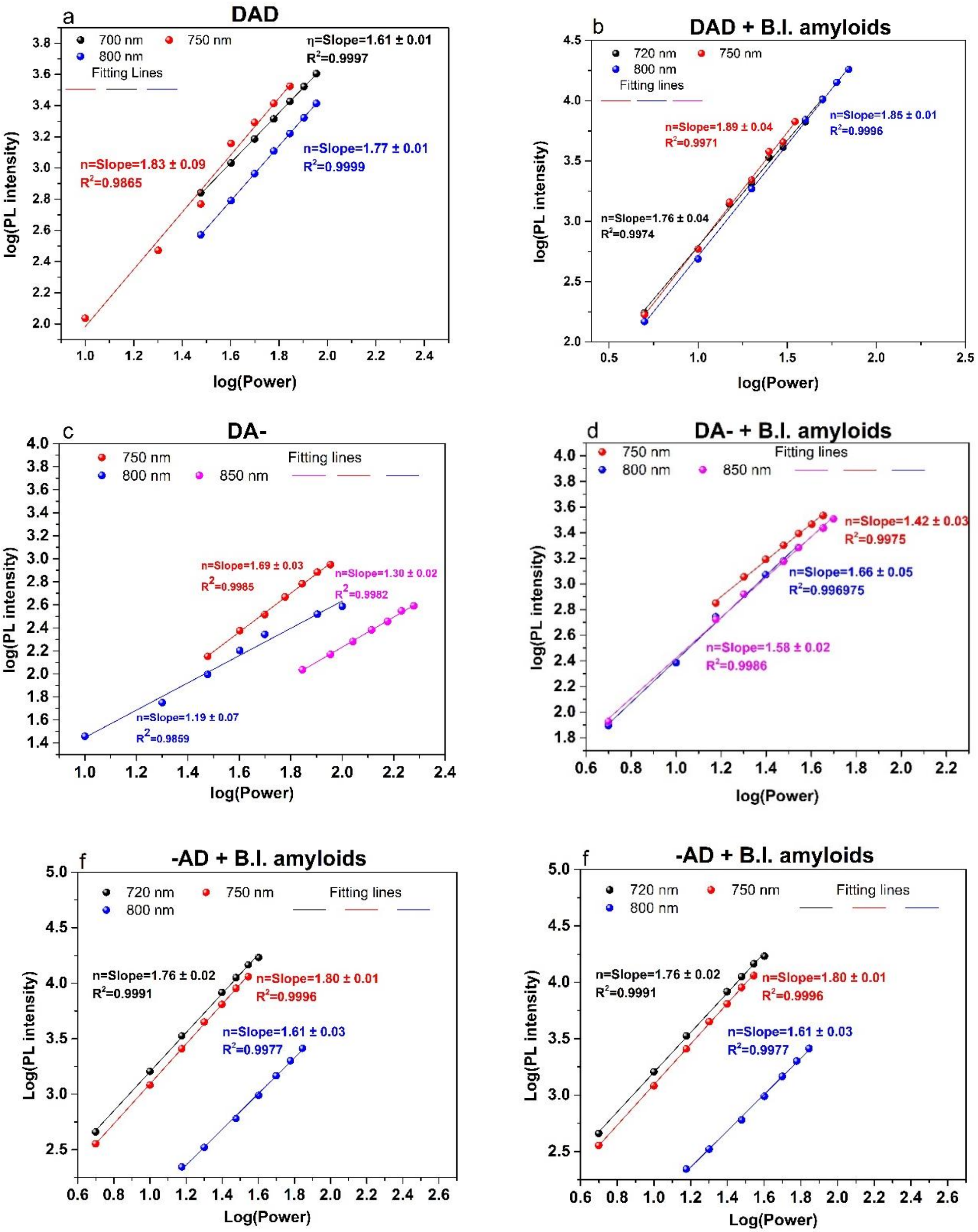

Figure S10. Log of the measured photoluminescence intensity (Log(PL intensity)) values plotted versus log of the incident laser power (log(Power)) for the studied free dyes: DAD (a), DA- (c), -AD (e) and upon the binding of bovine insulin (B.I.) amyloids to: DAD (b), DA- (d), and -AD (f).

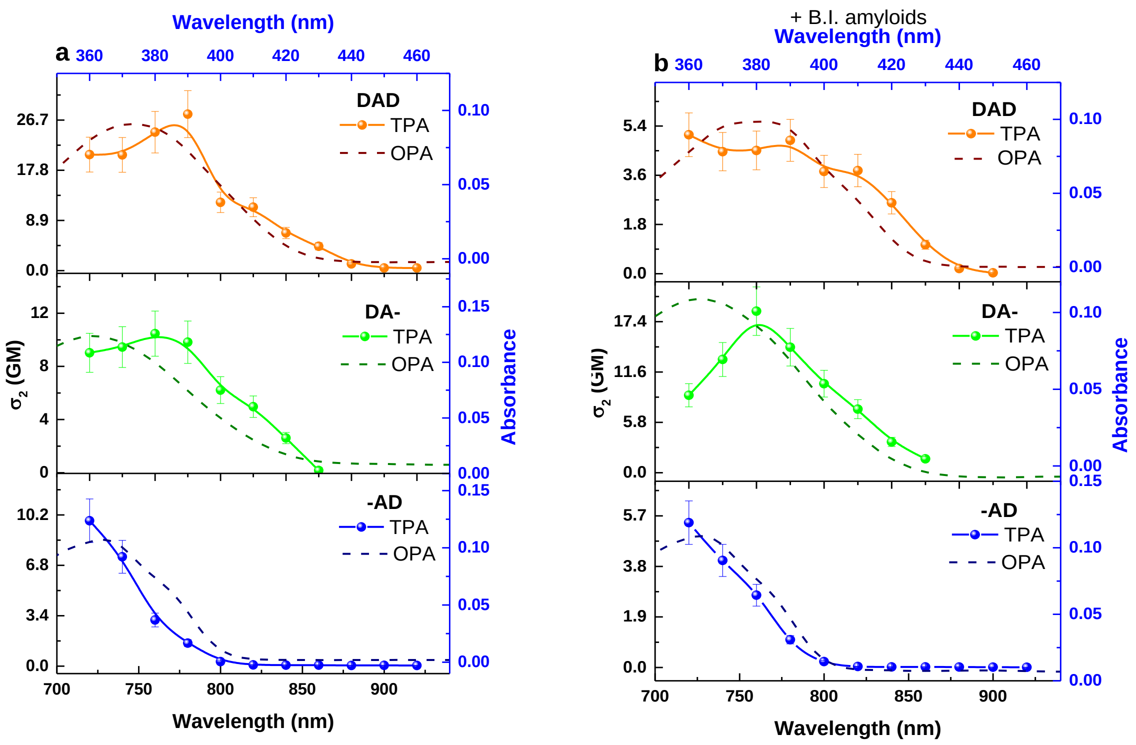

Figure S11. Two-photon absorption spectra with the corresponding one-photon absorption (1PA) spectra (a) free dyes in H_2O/DMSO mixture, (b) dyes with BI amyloids. Concentration of amyloids was chosen to provide the strongest one-photon fluorescence enhancement of each dye.

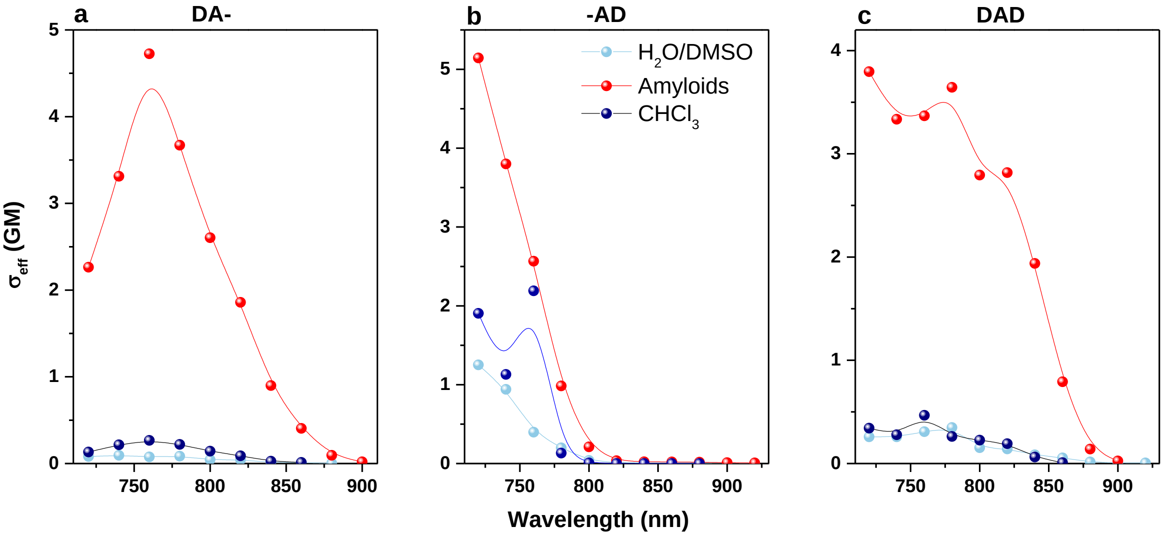

Figure S12. Effective two photon absorption cross-section for dyes in different solutions: $CHCl_3$, H_2O/DMSO, Amyloids. Concentration of amyloids was chosen to provide the strongest one-photon fluorescence enhancement of each dye. Uncertainty of values ±15%

Table S6. Calculated two-photon absorption cross sections (σ_2, [GM]). Two-photon absorption wavelengths (λ, [nm]) are given in parentheses. The results correspond to $S_0 \rightarrow S_1$ electronic transition.

DYE	σ_2[GM] (λ [nm]) chloroform	σ_2 [GM] (λ [nm]) water
**DA-**	47 (687)	57 (699)
**-AD**	12 (664)	29 (690)
**DAD**	5 (690)	11 (684)

References:

(1) Rybczynski, P.; Bousquet, M. H. E.; Kaczmarek-Kedziera, A.; Jedrzejewska, B.; Jacquemin, D.; Osmialowski, B. Controlling the fluorescence quantum yields of benzothiazole-difluoroborates by optimal substitution. *Chemical Science* **2022**, *13* (45), 13347-13360. DOI: 10.1039/d2sc05044g.

(2) Ran, C.; Wang, P.; Yang, J.; Zhu, B. Differentiating Aβ40 and Aβ42 with a small-molecule fluorescence probe. *Alzheimer's & Dementia* **2020**, *16* (S4), e041227. DOI: 10.1002/alz.041227

(3) Kuznetsova, I. M.; Sulatskaya, A. I.; Uversky, V. N.; Turoverov, K. K. Analyzing Thioflavin T Binding to Amyloid Fibrils by an Equilibrium Microdialysis-Based Technique. *PLOS ONE* **2012**, *7* (2), e30724. DOI: 10.1371/journal.pone.0030724.

(4) Mishra, R.; Sjölander, D.; Hammarström, P. Spectroscopic characterization of diverse amyloid fibrilsin vitro by the fluorescent dye Nile red. *Molecular BioSystems* **2011**, *7* (4), 1232-1240, DOI: 10.1039/C0MB00236D.

(5) Tokunaga, Y.; Sakakibara, Y.; Kamada, Y.; Watanabe, K.-i.; Sugimoto, Y. Analysis of Core Region from Egg White Lysozyme Forming Amyloid Fibrils. *International Journal of Biological Sciences* **2013**, *9* (2), 219-227, DOI: 10.7150/ijbs.5380.

(6) Vaiana, A. C.; Neuweiler, H.; Schulz, A.; Wolfrum, J.; Sauer, M.; Smith, J. C. Fluorescence Quenching of Dyes by Tryptophan: Interactions at Atomic Detail from Combination of Experiment and Computer Simulation. *Journal of the American Chemical Society* **2003**, *125* (47), 14564-14572. DOI: 10.1021/ja036082j.
